# The Effects of Radiation Therapy on the Ocular Apparatus: Implications for Management

**DOI:** 10.3390/cancers17162605

**Published:** 2025-08-08

**Authors:** Frank J. Arturi, Danielle Arons, Nicholas J. Murphy, Catherine Yu, Drishti Panse, Daniel R. Cherry, Kristin Hsieh, Julie R. Bloom, Anthony D. Nehlsen, Lucas Resende Salgado, Kunal K. Sindhu

**Affiliations:** 1 College of Medicine, SUNY Downstate Health Sciences University, 450 Clarkson Avenue, Brooklyn, NY 11203, USA; frank.arturi@downstate.edu; 2 Department of Radiation Oncology, Icahn School of Medicine at Mount Sinai, New York, NY 10029, USA

**Keywords:** uveal melanoma, ocular complications, central nervous system, head and neck cancer, radiotherapy, radiation oncology, organ-at-risk

## Abstract

Radiotherapy is utilized in the treatment of various cancers of the central nervous system and head and neck. Given the high concentration of organs-at-risk in this region, care must be exercised to minimize the dose delivered to these structures. Studies have shown that excessive radiation exposure can adversely impact the eyes, potentially resulting in the loss of their function. For instance, radiation doses greater than 50 Gy have been shown to increase the incidence of retinopathy, and radiation doses as low as 0.5 Gy have been shown to induce cataract formation. In this review, we discuss the ocular complications of radiotherapy used in the treatment of cancers of the central nervous system and head and neck. We then transition to potential strategies to spare the eyes during radiotherapy in an effort to reduce the rates and severity of ocular complications and preserve vision.

## Simple Summary

Radiation can be used to treat cancers of the brain and head and neck. With many important organs-at-risk in this area, radiation therapy courses must be planned meticulously to ensure that an excessive amount of radiation is not delivered to these sensitive structures. Excessive radiation doses to the ocular apparatus, for example, can impact vision and irreparably harm a patient’s quality of life. In this review, we discuss the potential ocular complications of radiation therapy and the strategies to spare the eyes and their surrounding structures from excessive exposure.

## 1. Introduction

Radiotherapy (RT) is utilized in the treatment of cancers of the central nervous system (CNS) and head and neck. By inducing double-stranded breaks in the deoxyribonucleic acid (DNA) of malignant cells, radiotherapy can effectively kill cancer cells [1]. However, in the process, radiotherapy may also affect noncancerous, healthy tissues. The brain and pharyngeal axis, in particular, have a high concentration of crucial organs-at-risk (OARs). Thus, practitioners must exercise caution in the treatment of tumors in these areas with radiotherapy in order to minimize damage to these tissues.

The ocular apparatus, including the eye, lacrimal gland, lens, optic nerves, and optic chiasm, constitutes one such set of OARs, and its function can be compromised by radiotherapy. Ocular complications of radiotherapy include, but are not limited to, dry eye syndrome [2], cataracts [3], and blindness. Given the importance of the ocular apparatus in maintaining quality of life, limiting excess dose to its structures is of the utmost importance. In this review, we discuss the structure of the eye, the use of radiotherapy to treat cancers of the CNS and head and neck, and the impacts of radiotherapy on various parts of the eye. Additionally, we review methods by which radiation oncologists can mitigate the risk of ocular complications, including by utilizing dose constraints, fractionation, shielding techniques, plaque brachytherapy, and contemporary external beam radiation therapy (EBRT) techniques like intensity-modulated radiation therapy (IMRT) and proton beam therapy (PBT).

## 2. Methods

We conducted a literature review using PubMed and Google Scholar. Search terms included anatomical terms (cornea, retina, lens, lacrimal gland, optic nerve, optic chiasm, ciliary body, and iris) and radiotherapy mitigation methods (external beam radiation therapy, proton beam therapy, eye plaque brachytherapy, and radiation eye shields). Other search terms employed included the alpha/beta ratio, series and parallel organs, and fractionation. Members of the research team screened relevant articles for inclusion in this review.

## 3. Structure of the Eye

**Figure 1:**
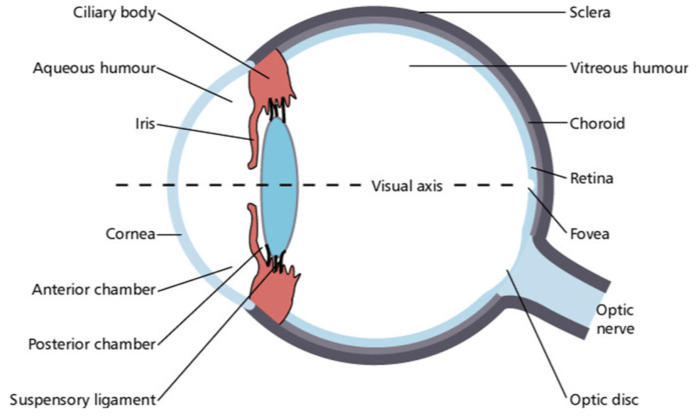
A sagittal view of the human eye, including its respective layers and chambers [10].

A thorough understanding of ocular anatomy and physiology is necessary to inform practitioners’ efforts to preserve the long-term function of the eye when delivering radiotherapy (see Figure 1). The human eye comprises three layers [4]. The outermost layer primarily functions to protect the internal structures of the eye and consists of the sclera and the cornea, which is responsible for three-fourths of the total refractive power of the eye [5,6]. The middle uveal layer consists of the iris, ciliary body, and choroid, and the interior layer is the retina [4,7].

The human eye may also be functionally compartmentalized into three chambers: the anterior chamber, the posterior chamber, and the vitreous chamber [4]. The anterior chamber is bordered anteriorly by the cornea and posteriorly by the iris and lens [8]. The posterior chamber is delineated anteriorly by the iris and centrally by the lens [9]. The vitreous chamber is the largest of the three chambers and is located between the lens and the retina [4].

The afferent visual pathway requires light conduction through the cornea, anterior chamber, pupil, and lens [4]. Then, the light must cross the vitreous chamber and impact the retina, which transmits the visual signal to the brain via the optic nerve [11]. After exiting the eye, the left and right optic nerves intersect at the optic chiasm, which is located at the base of the hypothalamus. The optic chiasm lies near the third ventricle, the anterior communicating and anterior cerebral arteries, and the pituitary gland [12]. As a result, any pathology associated with these neighboring structures renders the optic chiasm vulnerable to compression, resulting clinically in visual field loss.

**Figure 2:**
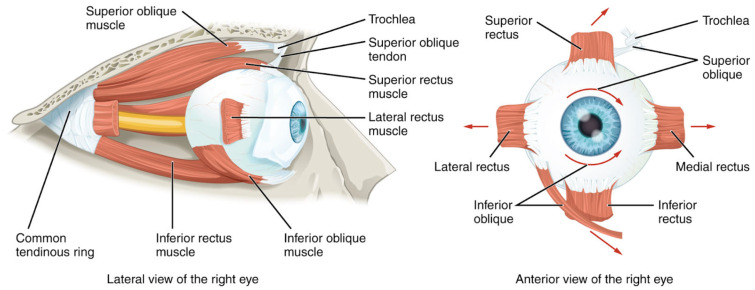
An axial view (**left**) and coronal view (**right**) of the human eye. The extraocular muscles of the eye are visualized [15].

In addition to mediating the visual pathway, the human eye is responsible for pupillary movements, conjugate gaze, and accommodation, each of which relies closely on ocular muscle architecture and its interaction with surrounding tissue. Movement of the human eye is controlled by the six extraocular muscles: the superior rectus muscle, the inferior rectus muscle, the medial rectus muscle, the inferior oblique muscle, the superior oblique muscle, and the lateral rectus muscle [13]. The three intrinsic muscles of the eye (the pupillary dilator muscle, the pupillary sphincter muscle, and the ciliary muscle) are responsible for pupillary dilation, pupillary constriction, and accommodation, respectively (see Figure 2) [4]. Of note, the ciliary muscle is housed within the ciliary body of the middle layer and contracts to mediate accommodation, the pathway that results in modified optical power to see objects clearly at varying distances [14].

Finally, the eyelid and tear film play essential roles in protecting the ocular surface and maintaining visual function. The eyelid comprises four layers, including skin and subcutaneous tissue, the orbicularis oculi muscle, the tarsal plate containing the meibomian glands, and the palpebral conjunctiva [16,17]. Together, these structures facilitate blinking, tear distribution, and mechanical protection. The tear film, a dynamic substructure formed by contributions from the lacrimal glands, goblet cells, and meibomian glands, consists of mucin, aqueous, and lipid layers [18,19,20]. It forms a critical barrier between the external environment and the corneal epithelium.

Radiation therapy, particularly when the eyelid, tarsus, or lacrimal gland is included in the treatment field, can disrupt tear film composition, reduce meibomian or lacrimal gland output, and impair blink mechanics. These changes increase the risk of exposure keratopathy, dry eye syndrome, and corneal ulceration [19,20,21,22]. Clinically, patients may experience foreign body sensation, photophobia, or visual decline. Ophthalmologic management often includes preservative-free lubricants, punctal occlusion, lid hygiene measures, or, in severe cases, surgical interventions such as tarsorrhaphy or amniotic membrane placement to protect the ocular surface [19,20,21,22].

In the context of tissue tolerance and response, it can be useful to organize the cells of an organ into functional units (FSUs), as introduced by Withers [23]. FSUs can be organized in series or parallel, much like an electric circuit [24]. With FSUs arranged in series, like the spinal cord, damage to one portion of the organ can cause inactivation further downstream [24]. In contrast, with parallel organs, function may be maintained, as the undamaged portion may function independently from the damaged portion. This can be seen in organs with subunits such as the kidneys. It is important to note that no tissues are purely serial or parallel [25]. Like other organs, components of the eye can be categorized as being serial or parallel. Consideration of the series or parallel nature and the effect of radiation on the FSUs of an organ can help to better inform dose constraints and treatment planning [26].

## 4. The Use of Radiotherapy in the Treatment of Cancers of the Head and Neck

### 4.1. Tumors of the Central Nervous System

Radiation therapy is widely used to treat cancers of the CNS. The most common use for radiotherapy in the treatment of primary CNS cancers is for patients with high-grade gliomas. Multiple randomized clinical trials, dating back to the 1970s, have established the efficacy of radiotherapy following biopsy or resection over best supportive care or chemotherapy alone [27,28,29,30]. BTCG 69-01, for example, showed that patients who received radiotherapy after surgery had improved overall survival as compared to patients who received chemotherapy or supportive care alone [27]. This benefit in overall survival with the addition of radiation therapy to surgery and chemotherapy has been confirmed in other studies [31,32,33]. As a result, today, adjuvant chemoradiation therapy following surgery or biopsy is the standard of care for patients with high-grade gliomas.

Radiation therapy is also used to treat some patients with low-grade gliomas. A large prospective study of patients younger than 40 years of age who had undergone gross total resection of their low-grade gliomas showed that 52% recurred within 5 years of surgery [34,35]. A randomized study, however, concluded that upfront adjuvant radiation therapy results in an improvement in progression-free survival without an accompanying benefit in overall survival [36]. Consequently, National Comprehensive Cancer Network guidelines recommend observation for patients with low-risk, low-grade gliomas. Adjuvant treatment with radiation and chemotherapy should be considered for all other patients [37,38,39].

Radiation therapy is also employed in the management of benign CNS tumors, including meningiomas [40]. Stereotactic radiosurgery, for example, when utilized to treat small meningiomas, is known to provide 10-year recurrence-free survival rates approaching 95% [41,42]. For larger lesions and in the postoperative setting, fractionated radiation therapy may be used [43,44].

Finally, radiation therapy plays an integral role in the management of patients with brain metastases. In the 1990s, a landmark trial established the role of whole-brain radiotherapy (WBRT) in the treatment of brain metastases [45,46]. Concerns about neurocognitive toxicity have, in recent years, led to both advances in the techniques through which WBRT is delivered and an increased utilization in stereotactic radiosurgery; these innovations have allowed for the delivery of ablative radiation doses without compromising overall survival and with fewer neurocognitive side effects [47,48,49,50,51].

### 4.2. Tumors of the Head and Neck

Radiotherapy is widely used in the treatment of cancers of the head and neck [52,53,54,55]. The options for the initial treatment of squamous cell carcinomas of the head and neck, for example, include surgery with or without adjuvant treatment, which consists of radiation therapy with or without concurrent chemotherapy, or definitive radiation therapy alone or with concurrent chemotherapy [56,57,58]. For patients receiving definitive chemoradiotherapy, American Society for Radiation Oncology guidelines advise the delivery of 70 Gy over 7 weeks to the gross primary tumor as well as clinically involved nodes. Lower doses are favored for clinically negative, at-risk areas. Altered fractionation may be employed in patients who are not receiving concurrent chemotherapy [58,59,60,61]. Nasopharyngeal cancers are treated primarily with RT, with or without chemotherapy, in the upfront setting.

### 4.3. Tumors of the Eye

Radiotherapy, in the form of either plaque brachytherapy or external beam RT, is the accepted standard of care for most localized uveal melanomas. This is based on randomized data in which plaque brachytherapy did not differ significantly from enucleation with respect to overall or distant metastasis-free survival for patients with small to medium-sized tumors [62]. Although both are acceptable approaches in certain cases, proton beam radiotherapy is also an option and may be preferred in cases in which the dose delivery characteristics of brachytherapy could prove suboptimal, such as those of larger tumors, those encircling the optic disk, involving extraocular structures, or in a non-operative candidate [63].

## 5. Impact of Radiation Therapy on the Eye

The impact of radiation therapy on orbital structures encompasses a wide spectrum of side effects, from dry eyes to decreased visual acuity. These effects correlate with radiation dose, the volume of orbital tissue in the radiation field, and the proximity of the primary lesion to the exposed orbital tissue [64]. Table 1 summarizes current research regarding toxicity, respective acuity, and available dose constraints with respect to the individual components of the eye.

### 5.1. Cornea

Radiation-induced corneal damage most commonly results from ocular dryness and tear loss post-treatment rather than direct corneal damage. However, radiation doses of 30 to 50 Gy have been reported to cause punctate corneal epithelial erosions, which may persist for months to years [65]. Corneal edema has been reported after radiation doses of 40 to 50 Gy due to endothelial dysfunction and the loss of the intact corneal epithelial barrier, which may result in photophobia, foreign body sensation, and/or decreased visual acuity [66]. Doses >60 Gy have been shown to result in corneal ulceration [65]. Acute toxicity has been shown to be more common than late toxicity, possibly due to stem cell loss, but there have been reports of permanent conjunctivalization of the cornea 3–4 years after RT [67]. A 2019 study by Lee et al. suggested a corneal maximum dose constraint of 51.8 Gy, with toxicity defined as requiring medical intervention [68].

Short-term treatments for corneal complications include topical lubrication for punctate epithelial erosions, hypertonic saline ointment, and topical steroids for improving corneal edema [66]. Long-term treatments include using bandage contact lenses or corneal transplant [66]. Other treatments that have been investigated include glued-on, gas-permeable contact lenses for radiation-induced keratitis [69], amniotic membrane transplantation [70], and ultraviolet radiation/riboflavin administration [71].

### 5.2. Lens

The lens of the eye is one of the most radiosensitive OARs to consider when administering radiation therapy. The principal detrimental consequence of RT on the lens is the development of cataracts [72]. The anatomy of the lens includes an outer membranous lens capsule that encases modified epithelial cells arranged as fibers, with an outer cortex of younger fibers and an inner nucleus composed of older fibers [73]. Each component of the lens is susceptible to cataract formation, which represents opacifications that obstruct light passage to the retina, thereby impairing vision. Patients may experience visual halos, a bothersome glare, difficulty reading or driving, or glaucoma [74]. In patients treated with RT, subcapsular, cortical, and nuclear cataracts are of most relevance, which are caused by fibrous metaplasia of the lens epithelium, cortical hydration between lens fibers, and the deposition of pigments like urochrome, respectively [74]. For mature cataracts, treatment usually involves surgery to extract the opacified lens and replace it with an artificial substitute [74].

Ainsbury et al. reviewed the existing literature surrounding the pathways by which low-dose ionizing radiation contributes to cataract formation. For many years, cataracts have been treated as a deterministic effect of radiation separated into acute and chronic (or fractionated) exposures with thresholds of 2 Gy and 5 Gy, respectively, for detection of lens opacity [72]. Cataracts that impaired vision were given thresholds of 5 Gy in the acute exposure setting and >8 Gy in the chronic/fractionated setting. More recent research, however, suggested that a dose of even 1 Gy could lead to cataract formation, which led the International Commission on Radiological Protection (ICRP) to set their threshold more conservatively at 0.5 Gy [75]. Thome et al. summarized all epidemiological data since 1956 and found doses below 0.5 Gy to be inconclusive [76]. Ainsbury et al. noted that this constitutes a simple, deterministic threshold, and many of the proposed mechanisms elucidated in the literature provide evidence that protracted ionizing radiation exposure leads to a significant increase in cataract incidence [72]. The proposed pathways include DNA damage and misrepair; damage to proteins, lipids, and the extracellular matrix; changes in the expression of genes and proteins; telomeric effects; intracellular communication changes; and oxidative reactions. It is likely that a combination of these effects ultimately leads to cataracts, often with a decades-long latency period.

Uwineza et al. introduced the concept of “cataractogenic load”, which takes into account the contributions of ionizing radiation exposure and alterations in all of the biomolecules found in the lens (including proteins, lipids, and DNA), which result from the aging process, the environment, and an individual’s genetics [77]. One such type of molecule is crystallins, proteins that are found at high concentration in the lens and whose orderly structure is key to the transparency of the lens. Oxidative stress, which can result from the formation of reactive oxygen species (ROS) after exposure to ionizing radiation, damages crystallins and leads to the aggregation of the proteins and loss of their ordered structure, causing a loss of transparency of the tissue. This is an important consideration as many proteins found in the lens are as old as the lens itself, owing to the loss of organelles such as the nucleus, mitochondria, and endoplasmic reticulum during the differentiation of lens fiber cells, which is required to ensure lens transparency. As the lens is a closed system involving organelle-free cells, this protein damage can accumulate over time, especially as protective mechanisms involving the glutathione redox cycle, superoxide dismutase, and peroxiredoxins decrease with age and thus allow the accumulation of ROS [78,79].

The Pediatric Normal Tissue Effects in the Clinic (PENTEC) study provides dose data corresponding to ophthalmologist-diagnosed and self-reported cataract formation. The risk of self-reported cataract formation was noted to be 5% at a mean dose of 12 Gy and 50% at a mean dose of greater than 40 Gy. The risk of ophthalmologist-diagnosed cataract formation, meanwhile, was 50% at a mean dose of 9 Gy. The data also notes that the age of pediatric patients does not appear to affect the risk of radiation-induced cataract formation, though children may potentially be at greater risk than adults (or, as noted, are screened more effectively) [80].

### 5.3. Lacrimal Glands

Radiation exposure can lead to dry eye syndrome (DES) via damage to Meibomian glands and acinar cells in lacrimal glands [2]. A retrospective review by Bhandare et al. found that, of 78 patients treated for primary head and neck tumors with external beam radiation therapy (EBRT) (doses ranging from 20 to 70 Gy), 51.2% developed severe DES that led to visual compromise [81]. Of note, none of the patients who received doses < 35 Gy developed DES, but all patients who received doses >65 Gy experienced severe DES. Radiation-induced DES did not vary across different patient demographics; age, gender, and the use of chemotherapy were not associated with a difference in DES development [81]. Similarly, a retrospective review by Nuzzi et al. of 25 patients who received EBRT for head and neck primaries demonstrated that 76% of patients developed DES, with the highest incidence in patients who received a maximum eye dose higher than 15 Gy [82].

Similar effects on the lacrimal glands were seen with WBRT in a prospective study conducted by Wang et al. Of the 100 included patients (doses ranged from 25 to 40 Gy), the median lacrimal dose was 25 Gy and the median lacrimal volume receiving 20 Gy (V20Gy) was 79% [83]. Of the 54 patients evaluable at 1 month, 32% and 24% had a ≥ 1 point increase and ≥2 point increase, respectively, in their Subjective Evaluation of Symptom of Dryness (SESoD) scores [83]. The proportion of patients with ≥1 point SESoD increase was 46% vs. 15% for patients with a lacrimal gland V20Gy ≥ 79% vs. < 79%, respectively, suggesting a dose-dependent relationship contributing to DES [83]. This was also noted in a prospective study of 90 patients who received EBRT (most received 46–55 Gy) for head and neck cancers by Soni et al. Overall, 53.3% of patients developed DES. A Pearson coefficient correlation of 0.987 suggested a strong correlation between higher radiation doses and the development of DES. Of patients who received 25–35 Gy, 1/6 (16%) developed DES, versus 9/13 (69%) patients who received 66–75 Gy. Some patients had symptoms as early as 1 week after completing radiation therapy. Interestingly, 9 patients in the study reported that they were asymptomatic despite having moderate-to-severe DES on physical exam. Patients with tumors that were closer to the orbit had higher rates of DES as compared to those with tumors that were located further away [84].

First-line treatment for DES is ocular lubricant (artificial tears). While this may provide symptomatic relief, it does not treat the underlying cause of DES. Additional treatments are currently under investigation. For example, a preclinical study by Kim et al. found that alpha-lipoic acid, by downregulating expression of NFAT-5 and thus, leading to less cell apoptosis in lacrimal gland cells in rats, could potentially have a role in both the prophylaxis and treatment of DES [85].

Laboratory work has also examined the cellular mechanisms by which radiation leads to DES. Eid et al. investigated the dose-dependent structural changes to lacrimal glands after exposure to radiation therapy using transmission electron microscopy (JEOL100 CXII, Tokyo, Japan). They found that even with minimal radiation exposure, lacrimal glands experienced injury to several cellular organelles, as well as damage to nearby blood vasculature and nerve endings. Loss of surface microvilli in intralobular ductal epithelial cells was seen and may be the mediator for early secretory granule retention, thereby leading to lacrimal gland toxicity [86].

### 5.4. Optic Nerves

With respect to the optic nerves, the 2010 Quantitative Analysis of Normal Tissue Effects in the Clinic (QUANTEC) data provides a summary for 3D conformal radiation therapy (3D-CRT) and stereotactic radiosurgery (SRS). For both treatments, the paper lists an endpoint of optic neuropathy. For 3D-CRT, the risk of optic neuropathy is generally low, at under 3% if the maximal dose (Dmax) is <55 Gy; however, it increases to 7–20% if the Dmax >60 Gy. For SRS, the Dmax is lower at <12 Gy, with a complication rate of <10%, due to the concentrated delivery of radiation [87]. Additionally, the PENTEC data provides dose constraints for the optic nerve and chiasm of 57 Gy, corresponding to a 5% risk of optic neuropathy, and 64 Gy, corresponding to a 50% risk of optic neuropathy [80].

Radiation-induced optic neuropathy (RION) presents as a painless, irreversible loss of vision. A retrospective study by Bhandare et al. that included 273 patients who received head and neck radiation with and without concurrent chemotherapy reported RION in 9% of patients. These patients were treated with one or two daily fractions at 1.1–1.2 Gy. Mean optic nerve doses in patients who developed RION were reported to be 67 Gy. This study found the risk of RION increased with age and hypofractionation, with no associated risk of RION in patients who received adjuvant chemotherapy [88].

While uncommon, RION may also occur in patients who receive lower doses of radiotherapy that are generally considered safe (<55 Gy for conventional RT, <8–10 Gy for SRS) [89]. Doroslovački et al. evaluated 18 such patients. A subgroup analysis suggested, however, that comorbid factors, like hypertension and smoking, may increase the risk of RION [89]. A review by Kinaci-Tas et al. found a RION prevalence of 2.0% across 70 examined studies. This figure varied significantly by the radiation dose administered: the prevalence was 4.5% and 1.7% at prescribed tumor doses of >50 Gy and <50 Gy, respectively. Of the half of the studies that reported the time to diagnosis after irradiation, the mean time was 36 months (range 3–108 months) [90]. Additionally, Li et al. conducted a retrospective review of 514 patients with chordoma or chondrosarcoma of the skull base treated with photon or proton radiotherapy. They showed that the cumulative incidence of RION was 1% and 5.8% in patients who received <59 Gy and ≥60 Gy, respectively, to the optic pathway. They also found older age, female gender, and higher maximum point dose to the optic pathway to be significant risk factors for the development of RION [91].

Unfortunately, there are currently no standard treatments for RION. Yu et al. conducted a systematic review and meta-analysis of studies conducted for the prophylaxis and/or treatment of RION. They found that intravitreal anti-VEGF injections and early hyperbaric oxygen therapy show the most promise as potentially effective treatments. Intravitreal anti-VEGF injections may also be an effective method of prophylaxis. Systemic anticoagulation, bevacizumab, and corticosteroids were not found to be effective treatment methods for RION [92].

To investigate the pathophysiology of RION, Yang et al. studied tree shrews and rats after receiving 20 Gy in two fractions using longitudinal manganese-enhanced MRI. Scans 30 weeks after irradiation showed a significant decrease in motor proteins, including cytoplasmic dynein-1, kinesin-1, and kinesin-2. Levels of proteins involved in axonal cytoskeleton structure, in contrast, including α-tubulin, β-tubulin, and SMI 31, were not statistically different. The authors concluded that a decline in axonal transport secondary to damage of motor proteins is responsible for the development of RION [93].

### 5.5. Retina

Retinopathy is a late effect of radiation that occurs in the months to years following treatment [90]. Radiation exposure to the retina leads to a loss of vascular endothelial cells through direct DNA damage and indirect damage via cytotoxic ROS and inflammation. Retinal vascular endothelial cells have a slow cell turnover, which is likely related to the delayed onset of radiation retinopathy. As these vascular cells die, small retinal capillaries become occluded, thereby leading to ischemia and ultimately retinal degradation and late-stage neovascularization. Clinically, this presents as partial or complete visual field deficits [94]. By contrast, the photoreceptors, which do not replicate, are inherently radioresistant [90,94,95,96,97].

Kinaci-Tas et al. systematically reviewed 78 studies between 1983 and 2022 of patients who received radiation therapy for tumors of the brain and head and neck. They found a pooled retinopathy prevalence of 6%, which was noted to be observed at prescription doses of >50 Gy. The prevalence of retinopathy was significantly higher (up to 70%) in studies of patients who underwent radiation therapy for nasal cavity carcinoma, likely due to the close proximity of these tumors to the retina and surrounding ocular structures. Twenty of the cited studies reported a diagnosis time, with a median of 39 months (range: 8–111 months) [90].

Additionally, Shen et al. reviewed 11 studies focused on pediatric ocular toxicities secondary to radiotherapy from 1980 to 2021, four of which reported on retinopathy. Using this data, they developed a predictive model that showed that maximum retina doses of 40 Gy and 62 Gy were associated with a less than 5% and a 50% risk of radiation retinopathy, respectively. The authors also recommended that this schema should be applied to the macula, as opposed to the entire retina, in the era of conformal radiation therapy as the macula is preferentially affected by radiation exposure [97].

Furthermore, a 2023 study by Zemba et al. investigated the role of various radiation modalities on ocular toxicity in the treatment of uveal melanomas. They included 78 articles from 1996 to 2021 and analyzed several complications, including retinopathy, which was differentiated into the reported complication rates by Ru-106 brachytherapy (20–53%), I-125 brachytherapy (10–62.8%), proton beam therapy (23–68.1%), and stereotactic radiosurgery (5–44%). Risk factors associated with each were also delineated, including higher doses to the tumor apex, increased tumor thickness, younger age, and higher radiation doses when using brachytherapy. The risk factors associated with external beam modalities were the presence of a tumor in the macular region, a reduced distance between the tumor and optic disk, diabetes mellitus, younger age, and radiation doses > 14.9 Gy. They noted that all complications were more common in younger patients, except for the development of cataracts [96]. Lastly, the PENTEC data provides a max dose of 42 Gy, corresponding to a 5% risk of retinopathy, noting that a higher fraction size corresponds to a greater risk of retinopathy [80].

Radiation retinopathy is a chronic condition. Unfortunately, a significant number of patients who develop this condition ultimately lose their vision in the affected eye. However, some treatments do exist, with varying degrees of success, including laser therapy to control neovascularization [98], corticosteroids [99], hyperbaric oxygen [100], pentoxifylline [101], and anti-VEGF therapies to mitigate neovascularization [102].

### 5.6. Pediatric Considerations

The developing eye is particularly susceptible to radiation-induced injury. Radiation exposure can disrupt the normal development of ocular structures and their surrounding orbital bones in children, potentially leading to facial asymmetry, enophthalmos, microphthalmia, and/or orbital hypoplasia. Additionally, damage to the lacrimal system, lens, and retina may present earlier, and progress more rapidly, in pediatric patients. Although PENTEC data offer guidance for pediatric dose constraints, the full spectrum of late effects in children remains incompletely characterized, particularly in the context of modern modalities like proton therapy. Thus, extra care must be taken in the treatment planning process to minimize radiation doses to critical ocular structures in children. Long-term ophthalmologic surveillance to monitor for delayed complications is essential.

## 6. Strategies to Mitigate Ocular Radiotherapy Complications

As described above, the structures of the eye have varying sensitivity to radiotherapy. Therefore, multiple approaches have been utilized to mitigate ocular radiotherapy toxicities, including using fractionation, eye shielding devices, constraining or excluding the eye or certain structures of the eye as OARs, and utilizing different radiation modalities. Table 2 summarizes the advantages and disadvantages of the different mitigation methods.

The α/β ratio describes a tissue’s response to fractionation, with higher values indicating sensitivity to total dose and lower values indicating increased sensitivity to fraction size [103]. A lower α/β ratio is typical of late-responding tissues, such as those in the eye, and suggests a greater capacity for tissue repair between smaller fractions [104]. α/β values for ocular structures are limited and variable in the literature. Vernimmen and Slabbert found no meaningful result for the optic chiasm, while Quashie et al. reported α/β values of 0.55 Gy (linear-quadratic-linear model) and 0.30 Gy (linear-quadratic model) for the optic pathway [105,106]. By contrast, the esophagus—a more acutely responding tissue—was found to have an α/β ratio of 5.38 Gy (linear-quadratic-linear model) and 4.71 Gy (linear-quadratic model) [106]. When the α/β ratio of the tumor is greater than that of surrounding normal tissue, fractionation can offer therapeutic benefit [105]. For periocular tumors, the eye’s α/β ratio of less than 1 Gy is typically lower than that of the tumor, supporting the use of fractionation strategies that reduce the risk of long-term ocular toxicity [103,105,106].

Eye shields have been used to decrease the radiation dose to the globe. There are two main types of eye shields: the internal eye shield, which is placed behind the skin of the eyelid, and the external eye shield, which is placed over the skin of the eyelid [107]. There are benefits and risks associated with the two types of shields. The internal eye shield may contribute to an increased eyelid dose due to the backscatter of incoming radiation [108]. The external eye shield, in contrast, by offering increased patient comfort and a decreased risk of corneal tearing, may be preferred in some clinical scenarios, such as total skin electron radiation for patients with mycosis fungoides without evidence of disease in the eyelid [109]. The eye shield may be made from several materials, including lead and tungsten, that attenuate an electron or photon beam [110,111]. Advantages of using eye shielding include the reduction in dose to ocular structures and customizability (different-sized shields and shields with acrylic coating to reduce patient discomfort) [101,109]. Disadvantages include the potential of backscatter and inappropriate usage (for example, using off-the-shelf photon shields for electron shielding) [112].

Applying dose constraints to the eye and its substructures is key to decreasing radiation dose to these sensitive organs during radiotherapy planning and treatment. In 2022, Bisello et al. published an organized list of dose-volume constraints for OARs, including the eye and its substructures [113]. In addition, several radiation modalities may mitigate ocular complications secondary to radiotherapy. The two main types of radiotherapy are EBRT, which involves the delivery of radiotherapy to the body from an external source, and brachytherapy, which involves placing radioactive materials within the body.

Photon-based EBRT delivered via intensity-modulated radiation therapy (IMRT) allows for the conformation or shaping of therapeutic radiation delivered to the intended target by utilizing non-uniform radiation beams and inverse planning [114]. Consequently, it is especially beneficial in clinical scenarios in which a radiosensitive structure, such as the eye, is in close proximity to the selected target. Zabel et al. found an improved dose distribution and decreased mean and maximum doses to the optic nerves, chiasm, and orbits when using IMRT instead of forward-planned, conformal radiotherapy for patients with esthesioneuroblastoma [115]. Photon-based radiotherapy may also be delivered via stereotactic body radiotherapy (SBRT), a technique that delivers high doses of radiation in few fractions [116]. SBRT may be preferred for smaller, well-defined tumors. It may also be preferred for its short treatment time, requiring fewer individual treatments, compared to IMRT [117,118]. The radiation beams may be arranged so that they do not pass through the contralateral eye or the ipsilateral cornea, thereby mitigating overall ocular toxicities [116]. With radiation treatment, there is always potential for normal tissue to be irradiated. Photons pass through the target tissue, irradiating more normal tissue along their paths than protons.

Proton beam therapy (PBT) is another type of external beam radiotherapy that has a unique property of dramatic distal dose fall-off due to the inherent properties of heavy particle therapy [119,120,121]. Thus, PBT may minimize radiotherapy-related toxicities by better sparing healthy tissues near the intended target due to the depth-dose characteristic known as Bragg Peak. Hartsell et al. reported that PBT with 3-dimensional treatment planning allows for excellent clinical treatment volume coverage and relatively low radiation doses to the eyes’ anterior structures, resulting in minimal acute toxicity for patients with ocular melanomas [122]. PBT has thus been used for treating multiple types of tumors, including ocular [123], central nervous system [124], head and neck, and pediatric malignancies [125,126], and in the re-irradiation setting [125,127]. Some disadvantages of PBT include accessibility and cost, and the relative lack of clinical trials comparing IMRT and PBT, although this continues to change for the better [128].

Precisely targeting ocular structures during radiotherapy is essential, especially when using high-dose-per-fraction treatments and modalities such as proton beam therapy (PBT), in which small positional shifts can significantly alter the dose distribution. Eye motion can thus pose a challenge. Eye immobilization is typically achieved through a combination of patient-specific thermoplastic masks and visual fixation targets that prompt the patient to gaze steadily in a fixed direction. In pediatric patients or those unable to adhere to treatment protocols, general anesthesia may be required. In select cases, thick contact lens–like suction cups can be applied directly to anesthetized globes to further immobilize the eye, even under general anesthesia, during which extraocular motion may still occur. Additionally, retrobulbar anesthesia, while less common, may also be used in select cases to immobilize the eye, though such an approach is associated with an increased risk of complications like hemorrhage, globe perforation, and optic nerve injury, among others.

Plaque brachytherapy is used to treat cancers of the eye, such as ocular melanoma [129]. Plaques usually consist of a gold-alloy metal backing with radioactive seeds, commonly palladium-103 or iodine-125 [130]. The plaque can be customized to the specific anatomy of the patient and tumor, mitigating radiation exposure to normal tissue [131,132]. The plaque is placed on the eye surface, and left for several days [132]. This procedure may help mitigate toxicities given its short therapeutic range, thereby sparing surrounding healthy tissues [133]. The Collaborative Ocular Melanoma Study Group led a randomized multicenter clinical trial assessing the survival outcomes of patients with ocular melanoma, and found no survival differences between the patients receiving iodine-125 brachytherapy and those receiving enucleation [62]. Thus, brachytherapy is offered to patients with intraocular tumors to maximize eye and vision preservation [123,134]. Some disadvantages include the procedure of placing the plaque itself and related possible surgical complications, and the potential adverse effects of radiation on normal tissue.

## 7. Limitations

This review has several limitations. In general, most of the studies examining the ocular complications of radiotherapy have examined patients who were treated many years ago. In fact, there are few studies analyzing the effects of radiation on the eye delivered in the modern era and with newer treatment modalities, such as with proton therapy. With that said, physiological tolerances to radiation and proper dose constraints may not be as well established with newer treatment modalities. Additionally, more recent studies may not have had sufficient follow-up to fully evaluate the long-term ocular risks of radiotherapy. As such, the data that we currently have on this topic may not be of direct applicability in the modern era of complex radiotherapy.

## 8. Conclusions

When using radiotherapy in the treatment of cancers of the CNS and head and neck, care must be taken to minimize damage to the eye. Over the years, techniques, including shielding and the use of different radiation modalities, have been utilized to protect the eye from excess radiation. Recent work has analyzed dosimetry with respect to different radiation modalities, allowing for the development of proposed constraints for key OARs. Future work will be of great value in continuing to mitigate the risk of developing ocular complications and identifying treatments for patients who have experienced such toxicities.

## Figures and Tables

**Table 1:** Table summarizing current research, with ocular component, authorship, toxicity, acuity, and triggering dose (fractionated unless specified).

Title 1	Author	Toxicity	Acuity	Dose
Cornea	Lee et al.	“Requiring Medical Intervention”	Chronic	51.8 Gy
	Barabino et al.	Corneal Edema	Acute	40–50 Gy
	Barabino et al.	Corneal Ulceration	Acute	>60 Gy
	Barabino et al.	Epithelial Lesion	Acute (Rarely Chronic)	30–50 Gy
Lens	ICRP	Cataracts	Acute	0.5 Gy
	Ainsbury et al.	Cataracts	Acute	5 Gy
	Ainsbury et al.	Cataracts	Chronic	>8 Gy
Lacrimal Glands	Nuzzi et al.	Dry Eye Syndrome	Acute and Chronic	>15 Gy
	Bhandare et al.	Dry Eye Syndrome	Chronic	34 Gy
Optic Nerve	QUANTEC	Optic Neuropathy	Chronic	55–60 Gy,
				<12 Gy (SRS Single Fraction)
	Bhandare et al.	Optic Neuropathy	Chronic	<50 Gy
Retina	Kinaci-Tas et al.	Retinopathy	Chronic	>50 Gy
	Shen et al.	Retinopathy	Chronic	40–62 Gy

**Table 2:** Table comparing advantages and disadvantages of ocular radiotherapy mitigation methods.

Method	Mitigation Provided	Advantages	Disadvantages
Shielding	Physical barrier for normal tissue	Size and material customizability	Backscatter, inappropriate shielding
Dose Constraints	Decreasing dose to normal tissue	Limits risk of toxicity	N/A
IMRT	Improved conformality	Increased organ-at-risk sparing	Increasing planning time
Proton Beam Therapy	Unique depth-dose characteristic	Improved precision of beam	Cost, accessibility, relative lack of clinical trials
Plaque Brachytherapy	Short therapeutic range	Customizable to patient anatomy	Surgical complications
Fractionation	Dose delivery over multiple sessions	Minimize toxicity	Longer treatment duration

## Data Availability

No new data were created or analyzed in this study. Data sharing is not applicable to this article.
